# Religious Insanity
*"The Relations of Religion to what are called 'Diseases of the Mind.'" 8vo Pamphlet, pp. 41. Philadelphia, 1850.


**Published:** 1850-07-01

**Authors:** 


THE JOURNAL

OF

PSYCHOLOGICAL MEDICINE

AND

MENTAL PATHOLOGY.

JULY 1, 1850.

### Art. I.—RELIGIOUS INSANITY.*

An editor must be very unfit for his important office who cannot patiently submit to criticism, in whatever shape and from whatever quarter of the globe it may come. The author of the pamphlet before us has thought proper to criticise ourselves, his object being to expose a "vein of error" which is alleged to pervade the whole of an article on religious insanity, published in the second number of this Journal.

Nothing would give us greater satisfaction than to be set right in any erroneous opinions and doctrines we may hold or promulgate on so important a subject; and we would most gratefully acknowledge such a service. In the present case, however, we feel that we have no thanks to render. Our reverend critic will please to remember that the beat of the "drum ecclesiastic" meets on this side of the Atlantic, and we believe on the other as well, with no response amongst scientific men. Theologians may be admitted to be high authorities in the pulpit; but in our little court, in which insanity is so frequently discussed, they can only take the place which a sound inductive philosophy allots to them. Considered in this point of view, we cannot pay a compliment to our reverend critic, and say that he is "a Daniel come to judgment." Indeed, so far as the substance-matter of the pamphlet concerns us, it hardly demands a serious notice at our hands; there are, however, dangerous errors in it, which are not peculiar to the writer, but rather to his *class*, and it is on this account we think it right to occupy a page or two with a

* "The Relations of Religion to what are called 'Diseases of the Mind.'" 8vo Pamphlet, pp. 41. Philadelphia, 1850.

notice of our critic's criticism. In the following paragraph the author commences his onslaught!—

"The reviewer sets out in his examination of Dr. Ideler's book with the singular assumption, that 'the object and aim of revealed religion is to modify the earthly and false principles which natural religion includes and promulgates; and the whole of what is termed religious education ought to be conducted with the view of bending the deductions of the untutored mind to the truths of revelation, and, by a diligent circulation of sound doctrines, eradicate the false.' The passage is almost obscure enough to pass for a scintillation of transcendentalism. So far as we can comprehend it, we think it places natural and revealed religion in an attitude towards each other which their relation by no means warrants."—p. 2.

The fact is, that our reviewer commits the mistake of reading the term *natural religion*, used by us to signify *false religions founded upon instincts*, in the sense of *natural theology*, the result of the philosophic and cultivated intellect. Let our critic comprehend that we meant something altogether different: we had Phallus and Fetish worship in view, not Plato or Paley. Natural religion arises out of man's "carnal nature"—to use the phrase of our author—that is to say, from his instincts and propensities; and just as one or other of these are predominant in a nation, or people, so will one or other manifestation of "the carnal mind" predominate in its religion, as lust, revenge, &c.

We adverted, in the article criticised, to the frightfully destructive wars, and the inconceivable cruelties, caused and practised by inexorable power amongst Christian nations, with the object of rooting out heresy, promulgating dogmas, or defending a degenerate or erroneous faith. Reverting to this passage, our reverend pamphleteer observes:—

"This picture is dismal enough, one would think. But our reviewer is, not unnaturally, betrayed into another error more plausible, and, therefore, more dangerous. He evidently regards the heretic as a religious madman, to be cured, like other madmen, by a purgative. 'To the psychologist,' he says, '*this dismal view of our holy religion*' (mark the expression), 'becomes the more dismal when he finds, in scrutinizing the details of history, that often the heretic was only a religious madman, and that purgation by hellebore would have been the remedy for his heterodoxy rather than purification by fire. Knowing the all-engrossing nature of religion, and the intensity of the emotions and feelings that it excites, he is prepared, *à priori*, to expect every form of insane aberration from religious truth, and every form of religious truth, and every form of mysticism and fanaticism. How often religious excitement is mere animal excitement! How often religious insanity is excited by religion, and how often by functional or structural diseases of the cerebrum—

are important questions to solve, inasmuch as the solution involves, not only the discrimination of what is religious truth, but also the determination of the etiology and treatment of insanity in matters of religious belief and conduct.' We readily admit the absurdity and sin of attempting to 'remedy heterodoxy' by fire; and we do not doubt that the vagaries of many religious errorists have originated in some disorder of the physical system, which medical skill might have controlled."—p. 5.

Our critic then goes on, through several weary pages, to disprove what needed no argument against it—that the heretic is not *always* a madman, as if, in the above sentence, we had asserted and maintained so absurd a proposition. *Inter alia,* he remarks:—

"If, for example, the denial of the divinity of our Lord and Saviour Jesus Christ must be regarded as a heresy, we can scarcely suppose our author to mean that it could be cured by 'a dose of hellebore.' The great Apostle to the Gentiles was not a 'religious madman' when he sought authority from the high priest, and set out on his bloody mission to seize, bind, and persecute to death any disciples of the Lord whom he could find out at Damascus. The wonderful scene which occurred upon the journey was not fitted to restore a maniac to reason, though it was marvellously efficacious in transforming a blasphemer into a believer, and a persecutor into an apostle. The madness was in his heart. The disease was in his moral nature, and not in the structure or functions of his cerebrum." —p. 6.

Who ever has said or argued to the contrary ? Not we, assuredly. All we have asserted is this, that a perusal of the histories of religious wars and persecutions leads us to the conviction, that the *insane* on religious ideas and doctrines were *often* punished with death, and particularly the cruel death by fire. That our reverend critic does not deny, we apprehend; if he do, we re-assert the fact; but we beg leave utterly to repudiate the doctrine which he imputes to us, that all heretics are necessarily insane.

The author of this pamphlet uses the term *religion* as significant only of the Christian religion, whereas we use it in its general signification, and as inclusive of every form of religious system or doctrine, whether Christian or Pagan, true or false, orthodox or heterodox. The sophisms which this double use of the word enables our critic to enunciate are numerous. He contrives (as shown above) to represent us as asserting that *all* heretics are necessarily mad; he also imputes to us the opinion, that the Christian religion, in its perfect truth and purity, makes men insane! His reference to Mormonism and Millerism will show how unreasonably he writes on this point.

"Passing over the many instances of such erratic and fanatical extravagances which history records, and to some of which the review before us alludes, we will glance at two recent and notable ones, occurring among ourselves, that we may the better judge whether religion makes men insane, or whether it merely fails, in many cases, to bring them to their right mind; so that it may be said that they continue insane in spite of all that religion can do for them."—p. 7.

Our author thus describes the results of "Millerism":—

"A man named Miller (a Baptist minister, as it is said) professed to have had a revelation of the precise day on which the second advent of Christ would occur, and when his people would be called to rise and meet him in the air! He and his deluded apostles, or agents, went from town to town, and from house to house, 'leading captive silly women,' and imposing upon the credulity of the ignorant. So settled was the conviction of many minds of the truth of his predictions, that they arranged their worldly affairs in reference to it, as an ascertained event, and made no contracts extending beyond the designated day. Prosperous citizens sold their estates, and declined the ordinary avocations of life, that they might give themselves wholly to the business of preparation; and, as the eventful period drew nigh, many evinced the sincerity of their convictions by providing what they regarded as suitable apparel for an aërial flight; and some actually assembled in groups upon summits which might be supposed most favourable to an early and easy ascension! The dupes of the false prophet were counted by thousands. Scores were committed to insane asylums, who were crazed with excitement or disappointment."—p. 9.

If a physician were to say these people were "crazed" with *religious* excitement, could he, by any reasonable use of language, be charged with asserting that they were "crazed" by "*revealed* religion?" Yet our reverend friend vents much theological and virtuous indignation, as if we had actually asserted so absurd a proposition, and goes on, page after page, defending religion from this imaginary imputation. We have found it difficult to get at our critic's notion of what constitutes religious insanity, but he is clearly of opinion that that form of mental aberration is necessarily *caused* by religion; for he suggests as one of the results of his series of arguments, that "there is no such thing as religious insanity: *i.e.* it cannot be said of religion, as it can be of grief, or disappointment, or chagrin, *that it causes* insanity." *We*, our critic was well aware, used the term in a definite sense, to imply mental aberration on religious matters and things of every kind whatever. The Hindoo or the Mahometan, the Roman-catholic or the Protestant, may alike be the subject of the disease. We gave the particulars of a most instructive case of religious insanity, from Ideler's Work. This patient entertained, upon religious subjects, insane ideas, which regulated all his acts for a lengthened period. Amongst other insane notions and practices in matters of religion, we stated that he became convinced, after reading the announcement by Christ of the destruction of Jerusalem, that the world was coming to an end, and that Berlin would be destroyed by fire. Acting on this insane idea, he set forth, with a piece of wood as an amulet, to bless the houses of his friends, the hospital for invalided soldiers, churches, &c., and thereby preserve them from destruction. He walked before the house fixed upon to be blessed, murmuring, "In the name of God the Father, the Son," &c. Here are insane religious actions, excited by an insane religious idea —namely, as to our Lord's second advent—and yet our reverend critic "cannot divine" what there was in the predominant features of the case to give it the character of *religious* insanity—that is to say, denies that there was any mental aberration on religious subjects ! After this statement, we think our readers will not expect us to analyze the critic's criticism of the cases. He is evidently unacquainted with the simplest elements of mental pathology. We fear, however, that he is not much better acquainted with his duties to Truth, otherwise he would have spared many of his remarks on this point; for in the whole of our article we most sedulously and most carefully repudiated the doctrine, that religion, in the sense used by our critic, is the cause of insanity. We stated emphatically—"During the course of our experience, we have never seen a case of insanity which could be clearly traced to true religion." We particularly drew attention to "the important distinction which is to be drawn between these deranged affections of the mind, resulting from the influence of false religion upon the understanding, and the healthy effects of legitimate Christianity upon the feelings and actions of man." These passages cannot have escaped this reverend pamphleteer's attention, for he quotes them—with a view, however, of weakening their force by an unjustifiable insinuation. He must also have been aware when he undertook to prove that "*there is no such thing as religious insanity*," of the sense in which the term "religious insanity" was used by us, for he observes—

"In this discussion, we assume [!] that Dr. Ideler and his reviewer mean, by the term 'religious insanity,' *that form of mental alienation which manifests itself upon religious subjects*,—as when one conceives himself to be God or the Saviour, or takes a false or defective view of his personal relations to God and his revealed laws, as by conceiving himself to have committed the 'unpardonable sin,' &c."—p. 16.

The absurd self-contradiction which our critic here manifests, arises necessarily out of his sophistical style of argument; in the discussion, *he* assumes that by the term religious insanity is meant, *insanity caused by true religion*, and not mental alienation upon religious subjects—*our* definition of the term. It is undoubtedly necessary to his style of argument that the term should be used in a *double* sense; but we call our critic's special attention to the *fact*, that, in doing this, he has wilfully perverted our views and plagiarized our principles.

This style of controversy is not unusual amongst theologians of the class to which our critic belongs; and as its origin is rather an interesting psychological study, we will notice it in some detail, especially as the pamphlet before us will afford some useful illustrations. The education of the clergy necessarily leads to a special study of theology, and the study of theology, at least amongst protestants, and especially amongst the "evangelical" section, is in a great degree biblical. So long as science is left out of consideration, this *exclusive* study of biblical theology gives rise to few, if any difficulties; but when human learning is brought to bear on questions of religious faith and practice, the danger of limiting the studies of the theologian becomes fearfully manifested, by the distressing doubts that arise out of the apparent collisions between scientific and revealed truths—we say *apparent*, because we are satisfied the two classes of truths never can come into real antagonism, and never ought to be considered as antagonistic. These observations apply obviously enough to the truths of astronomy and geology, and it is scarcely necessary to remind our readers, that there are even yet not a few Christian clergy to be found, who consider the great and general principles of those sciences to be utterly opposed to the astronomy and cosmogony of the Word of God, and that they ought, therefore, to be rejected. Greater difficulties even than in these two instances arise, when the clergyman has to reconcile medical with biblical psychology, for although the facts are more common and obvious, the apparent antagonism is very much greater, and much more difficult to reconcile, inasmuch as the principles of medical psychology are more recondite, and the study of them more laborious. It is a principle of medical psychology that the brain is the organ of the mind, and that, in insanity, it is the brain which is diseased. But the biblical theologian finds no such principles laid down in the works he has to study; to him the mind presents itself as an immaterial essence, capable of acting, in this life, apart from the brain and independently of it; all his views are therefore powerfully biassed thereby, and medical psychology consequently appears to him, not only to be directly antagonistic, but, we fear, is utterly abhorred by many of the clergy as rank materialism, if not infidelity. Yet the *facts* remain and must be got over, and the easiest method of doing this, is to acknowledge them verbally, but ignore them practically. It is precisely the method which our author has repeatedly adopted. On two consecutive pages of his pamphlet, we have first the facts acknowledged, and a principle of medical psychology granted, as follows:—

"We do not doubt that the vagaries of many religious errorists have originated in some disorder of the physical system, which medical skill might have controlled."—p. 6.

Then we have the facts, "we do not doubt," denied, and a principle of theological psychology asserted.

"He [St. Paul] voluntarily yielded himself to the dominion of malignant passions, and waged a war of extermination against the new religion, and against all who were otherwise minded than himself towards its claims. It is substantially so with *every errorist* and every persecutor. They refuse to come to Christ that they may have life. They have not his spirit; and *their heresy, whatever form it may assume, is the issue of a corrupt heart, not the vagary of a weak or deranged brain.*"—p. 7.

The methods of ministering to a mind diseased which medical psychology teaches are, in like manner, acknowledged on the one page and ignored on the following. We stated in our former article, that when there was already a predisposition to cerebral disease in a religious family, a fatal mistake might be made if the religious sentiment was encouraged in *early* infancy and childhood, and the youth thereby rendered *precociously* religious. We denounced an *exclusively* religious training in such cases, but no reasonable construction of our language could warrant any one in asserting that we advocated no religious training whatever. Let us see how our reverend critic handles this important practical point in the education of youth. First he acknowledges the truth of the *medical fact*, and of the therapeutics:

"A predisposition to cerebral disease in any family, religious or irreligious, should put all its members and friends on their guard against any influences likely to develop it."—p. 32.

Then he turns to his biblical psychology, and repudiates both the fact and its application.

"The wisest man that ever lived seems not to have apprehended the danger which our author points out, for he clearly enjoins an early encouragement of the religious sentiment in that familiar proverb of his,—'Train up a child in the way he should go, and when he is old he will not depart from it.' The 'way' in which all religious parents would doubtless wish their children to go 'when they are old,' is the way of the godly. And to this end the wise man (very philosophically it must be allowed,) counsels them to turn their childish footsteps into that way; in other words, 'to encourage the religious sentiment' in early infancy and childhood, *without any exception in favour of cerebral infirmities, or predisposition to insanity*."—p. 34.

If our reverend critic means anything by this, it must be, that the same mental training should be adopted in every case, without any exception in favour of the child with a tendency to water on the brain, to cerebral inflammation, to epilepsy, to insanity, and the other dreadful diseases to which children of remarkable talent and precocious genius are peculiarly liable. But how disastrously foolish,—nay, cruel,—would such a proceeding be considered! We addressed ourselves to the treatment of *exceptional* cases—cases of disease, in fact; our author admits the exceptional cases, then perversely argues as if our observations applied to education in general, and, lastly, admits of no exceptional cases whatever, because—Solomon made no exceptions ! Our reverend brother critic argues as if the science and art of medicine were to be found in the Bible; he is not solitary in his opinion—the Mormons, like other fanatics, attempt to heal their sick "by faith and prayer;" but how fanatically and fruitlessly we need not say.

This disingenuous method of argument necessarily renders the controversialist who adopts it dissatisfied with himself. He feels his incapacity to discuss the scientific questions raised. The vast field of modern psychology (which includes the relations of the mind to its material organs) is to him a sealed book; he knows nothing of the physiology of the brain or nervous system, for *his* psychology ignores even their existence; he is ignorant of even the meaning of terms used in the discussion of questions in which he ought to take the lead; and he endeavours to escape from the undefined, uneasy sensations he experiences, by misrepresentations, word-splitting, and petty quibbling. Instead of gnawing at the chain of ignorance by which he is restrained and bound, he vents his anger and impatience on the truths with which he is unacquainted. Our critic amply illustrates this state of mind. Let the reader peruse the following passage, and then, referring to our original article, or to our previous remarks, consider whether there be any fair or reasonable grounds for an imputation so flippantly and irreverently thrown out,—irreverently, because the solemnly important interests involved ought to secure the serious consideration of the most thoughtless. Our critic thus writes of us:—

"Our reviewer would evidently be a stanch advocate for the modern theory on this subject. He would probably argue, that as the too early inculcation of the religious sentiment tends to produce religious insanity, the too early inculcation of moral sentiments tends, in like manner, to the production of moral insanity. Hence he must, in all due consistency, advise that, where there is a predisposition to 'cerebral disease,' the distinction between right and wrong, truth and falsehood, integrity and dishonesty, should not be too strenuously urged; and that where these qualities seem to be confounded, so that a man mistakes another's watch and pocket-book for his own, or breaks open his neighbour's house under a misapprehension of the rights of property, or shoots an heiress because she will not marry him,—he should be put upon strict diet, all excitement of his nervous system avoided, and care taken to divert his mind from the study of ethical subjects.

"We are not trifling."—p. 40.

Now, in the passage thus animadverted on by our critic, we have not asserted the general proposition that the too early inculcation of the religious sentiment tends to produce religious insanity. As to the special case under notice, we stated that "the most important predisposing cause was *the want of a* GENERAL *education*," and we added, as to the class of special cases, "the irregularity of development of the mental faculties that will necessarily arise out of an *exclusively* religious training, will as necessarily lead to *irregularity of life and conduct*, and the proverb be verified in the individual,—'A young saint, an old devil.'" We at the same time pointed out the study of mathematics as the best "check to a *morbid* and predominant action of the religious sentiment," with the caution, however, that the study of the exact sciences might "extinguish a *minimum* amount, and so lead to another, and perhaps, more destructive form of insanity—a form characterised by an utter abandonment of all religious and moral principles whatever, and by utter depravity," (p. 236.) It was thus we briefly showed how irregularity of development of other faculties might induce the moral depravity to which our critic so flippantly refers; how, under certain circumstances, the man of commanding intellect might possibly become desperately wicked; and, by implication, how important it was, that in such cases an early and diligent inculcation of religious principles should be the rule. Such was the doctrine we laid down, and which we illustrated by examples; and these are the views which our critic has so flagrantly misrepresented in the passage quoted.

We repeat, that in these remarks we advocate great principles, and combat great errors; principles of incalculable value to society; errors of most baleful influence on religion and mankind. The misrepresentations of an obscure critic, totally ignorant of the subjects on which he writes, are of importance only because he represents a class, whose incapacity for their great and solemn duties is only equalled by their presumption. This class, we trustingly believe, is diminishing in Europe as regards numbers and influence; a theological literature is coming into existence which endeavours to render science what she ought to be—the handmaid of true religion;* which yields up none of the great and glorious truths of our holy religion to a false and presuming philosophy, but strips a false and presuming philosophy of its pretensions; and which stems infidelity by the very means that cause it.

The substance of this pamphlet appeared originally (as we learn from a foot-note) in the "Biblical Repository and Princeton Review," for January, 1850. We are not acquainted with the names of the editor or conductors, but we sincerely hope that its tone and matter do not constitute the reflex of the theological mind of the United States. Religion and morals cannot but suffer from an advocate such as is its writer. The immorality of an advanced and intellectual civilization requires more powerful and more cultivated intellects than his to stem it; and we are not without a trusting confidence that an all-wise Providence is, even now, raising up on the Trans-atlantic continent men able and willing to fit themselves for this great work. These will study men's nature in *all* its relations; they will seek to know, in especial, those laws by which his mind acts in and through its wondrous organ—the brain; they will look at and examine the express "image of God" in its place in nature, as well as in its moral relations through the Bible; they will diligently endeavour to reconcile truths, by extending their sphere of intellectual labour into the principles and details of science. They will do this, satisfied of the Heavenly origin of scientific truth, for "every good gift and every perfect gift is from the Lord," and grateful that science enables them to do the more effectually their blessed Master's work.

---

### Art. II.—CAUSES, CURE, AND PREVENTION OF IDIOCY.

This subject is occupying at this moment much public and professional attention. We are rejoiced to be able to say so. The condition of the poor idiot has been sadly overlooked, at least in this country. Psychologists, however, are disposed to make ample

* We would particularly direct attention to Dr. Harris's "Pre-Adamite Earth."

